# Creasote in Nævus Maternus

**Published:** 1844-11-30

**Authors:** 


CREASOTE IN NÆVUS MATERNUS.

Dr. Thornton informs us that of all the applications he has tried in nævus, (telangiectasis,) the most effectual is creasote. He had treated three cases in the course of the year successfully with this substance. It is applied two or three times, daily, more or less diluted. Excoriation, ulceration and gradual disappearance of the nævus ensues; the cicatrix has always been smooth and sound.—*Lond. Med. Gazette, from the Medicinische Zeitung.*

